# Wild ungulates as sentinels of flaviviruses and tick-borne zoonotic pathogen circulation: an Italian perspective

**DOI:** 10.1186/s12917-023-03717-x

**Published:** 2023-09-14

**Authors:** Laura Grassi, Michele Drigo, Hana Zelená, Daniela Pasotto, Rudi Cassini, Alessandra Mondin, Giovanni Franzo, Claudia Maria Tucciarone, Martina Ossola, Elena Vidorin, Maria Luisa Menandro

**Affiliations:** 1 Department of Animal Medicine, Production and Health (MAPS), University of Padua, Viale dell’Università, 16, Legnaro, PD 35020 Italy; 2 Department of Virology, Institute of Public Health, Ostrava, Czech Republic

**Keywords:** Vector-borne zoonotic pathogens, Flavivirus, Wild ungulates, *Ixodes ricinus*, Molecular biology, Virus neutralization test

## Abstract

**Background:**

Vector-borne zoonotic diseases are a concerning issue in Europe. Lyme disease and tick-borne encephalitis virus (TBEV) have been reported in several countries with a large impact on public health; other emerging pathogens, such as *Rickettsiales*, and mosquito-borne flaviviruses have been increasingly reported. All these pathogens are linked to wild ungulates playing roles as tick feeders, spreaders, and sentinels for pathogen circulation. This study evaluated the prevalence of TBEV, *Borrelia burgdorferi* sensu lato, *Rickettsia* spp., *Ehrlichia* spp., and *Coxiella* spp. by biomolecular screening of blood samples and ticks collected from wild ungulates. Ungulates were also screened by ELISA and virus neutralization tests for flaviviral antibody detection.

**Results:**

A total of 274 blood samples were collected from several wild ungulate species, as well as 406 *Ixodes ricinus*, which were feeding on them. Blood samples tested positive for *B. burgdorferi* s.l. (1.1%; 0-2.3%) and *Rickettsia* spp. (1.1%; 0-2.3%) and showed an overall flaviviral seroprevalence of 30.6% (22.1–39.2%): 26.1% (17.9–34.3%) for TBEV, 3.6% (0.1–7.1%) for Usutu virus and 0.9% (0-2.7%) for West Nile virus. Ticks were pooled when possible and yielded 331 tick samples that tested positive for *B. burgdorferi* s.l. (8.8%; 5.8–11.8%), *Rickettsia* spp. (26.6%; 21.8–31.2%) and *Neoehrlichia mikurensis* (1.2%; 0-2.4%). TBEV and *Coxiella* spp. were not detected in either blood or tick samples.

**Conclusions:**

This research highlighted a high prevalence of several tick-borne zoonotic pathogens and high seroprevalence for flaviviruses in both hilly and alpine areas. For the first time, an alpine chamois tested positive for anti-TBEV antibodies. Ungulate species are of particular interest due to their sentinel role in flavivirus circulation and their indirect role in tick-borne diseases and maintenance as *Ixodes* feeders and spreaders.

**Supplementary Information:**

The online version contains supplementary material available at 10.1186/s12917-023-03717-x.

## Background

Vector-borne diseases are described as emerging infections due to their massive spread in recent decades. Tick-borne diseases (TBDs) and mosquito-borne diseases represent the most threatening vector-borne infections worldwide [1]. The rise of vector populations, together with the pathogens they may carry, is a deeply studied topic, but there are many aspects yet to be revealed [2–5].

The diffusion of TBDs is driven by several factors, mainly consequent to human activities. Modern agriculture has led to changes in land use leading to an increase in mountain abandonment, usually followed by natural reforestation, which is a frequently reported phenomenon in the Eastern Alps [6, 7]. In turn, land abandonment stimulates an increase in wild animals, which can boost tick presence [2].

Wild ungulates are abundant in northern Italy [8, 9] and are known as preferential hosts for tick feeding and reproduction, especially for *Ixodes ricinus* (*I. ricinus*) ticks [9, 10]. In Europe, as well as in northern Italy, *I. ricinus* is one of the most studied tick species due to its high competence in the transmission of zoonotic pathogens, including viruses, bacteria, and parasites, to humans [1, 11–13].

The most prevalent zoonotic agents transmitted by *I. ricinus* in Europe belong to the *Borrelia burgdorferi* sensu lato complex [14]. Other zoonotic and emerging pathogens transmitted by *I. ricinus* bite are tick-borne encephalitis virus (TBEV), *Rickettsia* spp., *Anaplasma phagocytophilum* (*A. phagocytophilum*), *Ehrlichia* spp., and *Neoehrlichia* spp. [15–18]. Currently, *Borrelia burgdorferi* sensu latu (*B. burgdorferi* s.l.) is the main zoonotic tick-borne pathogen in northern Italian regions [19], while TBEV [20], *A. phagocytophilum*, and *Rickettsia* spp. [15] have been only sporadically detected in humans, whereas *Ehrlichia* spp. and *Neoehrlichia* spp. [15]. have not yet been identified.

The epidemiological cycles of these infections involve wild ungulates that mainly act as tick feeders and spreaders in the environment, as well as reservoir hosts of *A. phagocytophilum* [21]. In contrast, *I. ricinus* acts as both a vector and reservoir for *Rickettsia* spp. [22], and with regard to the other infectious agents, different vertebrate species serve as reservoir hosts [21, 23, 24].

*Coxiella burnetii* (*C. burnetii*) has been identified in *I. ricinus*, although its vector capacity is still debated [25, 26]. It may cause disease in humans and livestock [27], and similar to domestic ruminants, red deer and wild boar are susceptible to *C. burnetii* and have been suggested to play a role in its maintenance [28].

Flaviviruses are currently the most widespread vector-borne zoonotic viruses in Europe and are transmitted by hard ticks (TBEV) or mosquitos (Usutu virus-USUV and West Nile virus-WNV). Both USUV and WNV are considered endemic in Italy first being detected in black birds (1996) and horses (1998), respectively [29, 30]. The epidemiological cycle of these flaviviral species includes *Culex* mosquitos and wild birds. Mammals, including humans, can be infected but are considered dead-end hosts [31–33].

Regarding TBEV, Italy is considered a low-risk country, and most cases of human infection are found in the northeastern regions [20]. The main vector is *I. ricinus* [34], but the detection of TBEV in ticks is challenging, time-consuming and expensive since the prevalence is restricted to small foci of viral circulation [24]. In areas where TBEV incidence is high in humans, prevalence in ticks rarely exceeds 1% [35, 36]. Recently, wild ungulates have been considered TBEV sentinels, noting that they seroconvert but usually do not develop disease, even though a fatal case has been recently described in a roe deer [34, 37–39]. In European countries, TBEV antibodies were found in several wild ungulate species [34, 40–42], with seroprevalence ranging between 0.74% in Finland and 63.5% in Poland. Despite the comparable clinical and epidemiological scenarios, fewer studies have investigated the seroprevalence in ungulates for WNV and USUV; seroconversion in asymptomatic animals was reported, suggesting the role of ungulates as sentinels in viral diffusion [36, 43, 44].

The involvement of wild ungulates in the epidemiology of all the abovementioned vector-borne pathogens is noticeable, and although several of these etiological agents are considered endemic to the alpine area of northern Italy, data derived from ungulate populations are currently scarce.

This research aimed to (i) assess the prevalence of a zoonotic virus (TBEV) and zoonotic bacteria (*B. burgdorferi* s.l., *Rickettsia* spp., *Ehrlichia* spp., *C. burnetii*) in wild ungulate blood and associated tick samples using biomolecular tools; (ii) identify the selected species and genospecies through sequencing; (iii) investigate the sentinel role of ungulates in flaviviral (TBEV, WNV, USUV) circulation by antibody detection; and (iv) evaluate any the statistical significance of the obtained prevalence and seroprevalence related to several variables of interest.

The zoonotic bacterium *A. phagocytophilum* was purposely excluded from this study since it was previously investigated in the same ungulate and vector population [21].

## Results

### Sample collection and molecular biology

#### Blood samples

A total of 281 blood samples were collected from culled wild ungulates in three different investigated regions: Lombardy (n = 105), Veneto (n = 67), and Friuli Venezia Giulia (n = 109). The species of ungulates included roe deer (*Capreolus capreolus*, n = 108), red deer (*Cervus elaphus*, n = 87), wild boar (*Sus scrofa*, n = 59), mouflon (*Ovis orientalis musimon*, n = 18), and chamois (*Rupicapra rupicapra*, n = 9). The DNA/RNA internal control was not detected in seven samples, which were excluded from the study; therefore, 274 blood samples were investigated for tick-borne pathogens (TBPs). Additional details are provided in Table 1.

**Table 1:** Details on ungulate species tested in the study divided by the region of origin

Ungulate species	Lombardy	Veneto	Friuli Venezia Giulia	Total
Roe deer	33	24	50	**107**
Red deer	44	22	17	**83**
Wild boar	17	12	29	**58**
Mouflon	9	4	4	**17**
Chamois	0	0	9	**9**
**TOTAL**	**103**	**62**	**109**	274*

*Seven samples tested negative at the internal control screening and were excluded from the analysis for TBP detection; thus, the analysed blood samples (n. 274) are less than the initial number of sampled animals (n. 281)

Three out of 274 (1.1%; 0-2.3%) blood samples collected from two red deer (from Lombardy) and a wild boar (from Veneto) were positive for *B. burgdorferi* s.l.. Due to the low quantity of bacterial DNA (Cq > 35), none of the samples were confirmed by PCR and sequencing. Three out of 274 (1.1%; 0-2.3%) blood samples collected from a roe deer (from Lombardy), a red deer and a wild boar (from Friuli Venezia Giulia) tested positive for *Rickettsia* spp. Sequencing analysis confirmed the detection of an unidentified *Rickettsia* spp. from roe deer blood, with only 96% identity with known rickettsiae. Furthermore, *R. helvetica* was identified from red deer, while positivity of the wild boar blood was not confirmed by PCR. All blood samples tested negative for *Ehrlichia* spp., *Coxiella* spp., and TBEV.

#### Tick samples

A total of 406 ticks were collected and extracted in pools of two ticks (n = 75) or individually (n = 256). Thus, the total number of screened tick samples was 331 (Table 2). All ticks were identified as *I. ricinus* and most of the tick samples consisted of adult females (n = 231) or males (n = 90), while few immature stages were found, i.e., nymphs (n = 6) and larvae (n = 4). Most of the ticks were collected from roe deer, red deer, and mouflons. All tick samples tested positive for the DNA/RNA internal control, showing no PCR inhibition.

**Table 2:** Results of tick-borne pathogen screening and species identification in *I. ricinus* tick samples

Host species	Tick samples^c^	*B. burgdorferi* s.l. n (%) (95%CI)	PCR *Borrelia*	*Rickettsia* spp. n (%) (95%CI)	*PCR Rickettsia*	*Ehrlichia* spp. n (%) (95%CI)	PCR *Ehrlichia*
Roe deer	168	15 (8.9) (4.6–13.2)^a^	5/15 *B. garinii* 6/15 *B. afzelii* 1/15 *B. valaisiana*	44 (26.2)^b^ (19.5–32.8)	17/44 *R. monacensis* 22/44 *R. helvetica* 2/44 *Ca.* R. mendelii	4 (2.4) (0.1–4.7)	4/4 *N. mikurensis*
Red deer	138	10 (7.2)^a^ (2.9–11.6)	2/10 *B. garinii* 1/10 *B. afzelii* 1/10 *B. burgdroferi* s.s. 1/10 *B. valaisiana*	37 (26.8)^b^ (19.4–34.2)	16/37 *R. monacensis* 11/37 *R. helvetica* 2/37 *Rickettsia* spp.	0	
Wild boar	2	1 (50.0) (0-100)	1/1 *B. afzelii*	2 (100.0)	1/1 *R. monacensis* 1/1 *R. helvetica*	0	
Mouflon	22	3 (13.6)^a^ (0–28.0)	2/3 *B. afzelii*	5 (22.7)^b^ (5.2–40.2)	1/5 *R. monacensis* 2/5 *R. helvetica*	0	
Chamois	1	0	-	0	-	0	
**TOTAL**	**331**	**29 (8.8)** **(5.7–11.8)**	**-**	**88 (26.6)** **(21.8–31.4)**	**-**	**4 (1.2)** **(0.0-2.4)**	

^a,b^ Statistical analysis was conducted at the host species level reporting at least 5 counts; ^a^ X^2^ = 1.06, P = 0.588; ^b^ X^2^ = 0.16, P = 0.923

^c^ Tick samples include both individual and pooled ticks (composed of two individuals)

Twenty-nine out of 331 (8.8%; 5.8–11.8%) tick samples tested positive for *B. burgdorferi* s.l. Amplicon sequencing identified the following species: *Borrelia garinii* (*B. garinii*) (7/29), *Borrelia afzelii* (*B. afzelii*) (10/29), *Borrelia burgdorferi* sensu stricto (*B. burgdorferi* s.s.) (1/29) and *Borrelia valaisiana* (*B. valaisiana*) (2/29); however, some amplicons from tick samples (9/29) did not yield good quality sequences, and thus, genospecies identification was not possible.

A total of 88 out of 331 (26.6%; 21.8–31.4%) of the tick samples were positive for *Rickettsia* spp. Two zoonotic species were identified, namely, *Rickettsia monacensis* (*R. monacensis*) (35/88) and *Rickettsia helvetica* (*R. helvetica*) (36/88). *Candidatus* Rickettsia mendelii (*Ca.* R. mendelii) was found in two samples from ticks feeding on roe deer.

Four out of 331 tick samples (1.2%; 0-2.4%) collected from roe deer tested positive for *Neoehrlichia mikurensis* (*N. mikurensis*). No other *Ehrlichia* species were identified.

The prevalence of *B. burgdorferi* s.l. and *Rickettsia* spp. in tick samples did not show any significant difference when considering the ungulate species from which the ticks were collected. All tick samples tested negative for *Coxiella* spp. and TBEV. More details and data on the host species from which positive ticks were collected are shown in Table 2.

Ten out of 331 samples (3%; 1.2–4.9%) were positive for more than one pathogen. The presence of concurrent bacterial species was confirmed by Sanger sequencing in 6 of 10 specimens: coinfection between *B. afzelii* and *R. helvetica* was found in 2 pooled samples, one pool of male ticks and one of females, and in 3 individually extracted ticks, one female and two males. One of the males also tested positive for a third pathogen, *N. mikurensis*. A different male tick tested positive for both *B. afzelii* and *R. monacensis*.

#### ELISA and virus neutralization for flaviviruses

A total of 111 serum samples originated from the Veneto (n = 46) and Friuli Venezia Giulia (n = 65) regions; no serum samples were available from Lombardy. All ungulate species were represented, namely, roe deer (n = 45), red deer (n = 31), wild boar (n = 23), chamois (n = 8), and mouflon (n = 4).

In total, 34 out of 111 blood samples tested positive by ELISA showing an overall anti-flavivirus seroprevalence of 30.6% (22.1–39.2%); in detail, 24 samples were positive, and 10 were borderline. All sampled species were positive, except for mouflon. The detailed seroprevalence of the tested species is shown in Table 3.

**Table 3:** ELISA and VNT test results of screened wild ungulate sera

Ungulate species	Tested sera	ELISA n pos (%;95%CI)	TBEV VNT n pos (%;95%CI)	USUV VNT n pos (%;95%CI)	WNV VNT n pos (%;95%CI)
Roe deer	45	17 (37.7; 23.6–51.9)	17 (37.7; 23.6–51.9)	0	1 (2.2; 0-6.5)
Red deer	31	8 (25.8; 10.4–41.2)	7 (22.6; 7.9–37.3)	0	0
Wild boar	23	8 (34.8; 15.3–54.3)	4 (17.4; 1.9–32.9)	4 (17.4; 1.9–32.9)	0
Mouflon	4	0	-	**-**	**-**
Chamois	8	1 (12.5; 0-35.4)	1 (12.5; 0-35.4)	0	0
**TOTAL**	**111**	**34 (30.6; 22.1–39.2)**	**29 (26.1; 17.9–34.3)**	**4 (3.6; 0.1–7.1)**	**1 (0.9; 0-2.7)**

The virus neutralization test (VNT) confirmed that most of the ELISA-positive sera were positive for TBEV (29/34), resulting in an overall prevalence of 26.1% (17.9–34.3%). Four ungulate species had TBEV antibodies: chamois, wild boar, roe deer and red deer (Table 3). Positive animals were mainly from the alpine and prealpine areas of both investigated regions. Of note, some were shot in a hilly area (altitude is approximately 170–180 m above see level) close to the flatlands. In addition, a roe deer tested positive for both TBEV and WNV.

Of the five TBEV-negative samples, one was negative for all investigated flaviviruses, while the remaining four were USUV positive. These were wild boars culled in a hilly area of the Friuli Venezia Giulia region; three were culled between June and October of the same year in different municipalities but in a range of 10–15 km^2^. Flavivirus antibody positivity did not show any statistical association with “species”, “sex”, “age”, and “season” variables. Detailed results of ELISA and VNT tests are available in Additional file 3.

## Discussion

The present study evaluated the presence and frequency of several vector-borne pathogens using molecular biology and serology. Except for *C. burnetii*, all investigated pathogens were identified through direct or indirect testing, highlighting the wide diffusion of both tick-borne and mosquito-borne infections in northern Italy.

Overall, the medical and veterinary importance of *I. ricinus* was confirmed by its high vector occurrence and remarkably frequent infection with zoonotic pathogens. According to the present results, wild ruminants (except for chamois) appear to be highly infested compared to wild boars, but they seem to play a minor role as reservoirs for tick-borne infectious agents. On the other hand, since most of the feeding *I. ricinus* were adults, the role of ungulates as tick amplifiers was confirmed. In addition, ungulates appear to be promising sentinels of flavivirus infections.

*B. burgdorferi* s.l. was detected in both blood (1.1%) and tick (8.8%) samples, although the low amount of *Borrelia* DNA (high Cq) in blood samples hampered further characterization. The low levels of bacteria are in accordance with previous investigations that outlined how wild ungulates can act as *Borrelia* dilution hosts, and thus, blood positivity is only sporadically identified without any apparent epidemiological relevance [45]. However, the role of artiodactyls is controversial: on the one hand, they represent an important feeding source for ticks; on the other hand, they have shown a limited contribution to the transmission of the Lyme spirochete, decreasing the spread of infection [45, 46]. Moreover, *I. ricinus* tick samples showed a noticeable prevalence of *Borrelia* spp. and several zoonotic species were identified. The detection of four genospecies (*B. garinii*, *B. afzelii, B. burgdorferi* s.s., and *B. valaisiana*) indirectly highlights the presence of suitable reservoirs in the study area, such as wild birds and small mammals, and confirms the endemicity of these pathogens in the study area. A previous study [23] conducted in northeastern Italy on questing ticks reported the same zoonotic species, although at a lower prevalence, and similar identifications were found in northwestern Italy [47, 48]. The prevalence reported herein is also higher than that described in Spain (2.3%) and Poland (3.3%) [49, 50], likely due to the diversity of the ecological niches in different areas.

Evidence of *Ehrlichia*, particularly *N. mikurensis*, was limited to the vectors, with a prevalence of 1.2%, in agreement with a previous study conducted in a nearby area on questing ticks [23]. Despite the recent discovery of *N. mikurensis*, it seems that rodents could be reservoirs, while wild ungulates may act as tick spreaders rather than amplifiers [51].

In contrast, *Rickettsia* spp. was detected in both blood samples and feeding ticks. The prevalence in blood was low (1.1%), and the detection of *R. helvetica* (in red deer) and an unidentified *Rickettsia* (in roe deer) is likely to be an occasional finding because wild ruminants are not considered reservoirs of infection [52]. In contrast, the prevalence in tick samples was high (26.6%). Most *Rickettsia* spp. were *R. monacensis* and *R. helvetica*, which are typically found in inland and continental areas [15]. A lower (Slovakia – 6.8%) and similar (Poland – 26.8%) prevalence in *I. ricinus* collected from wild ungulates was reported by other authors, reflecting the heterogeneity between habitats [12, 50]. Although these rickettsial species are considered pathogenic only in immunocompromised patients, a recent case described the onset of disease in an immunocompetent patient in Portugal [53].

Two ticks were positive for *Ca.* R. mendelii, which was first identified in 2016 in *I. ricinus* in Eastern Europe [54] and has been reported in *I. ricinus* questing ticks in Poland and the Czech Republic and in feeding ticks on migratory birds in Italy. The zoonotic potential of *Ca.* R. mendelii is still unknown [15, 55, 56].

*C. burnetii* was not found in blood and tick samples, whereas a prevalence of approximately 5% had been reported both in questing and wildlife-collected *I. ricinus* [25, 26]; other in vivo experimental studies demonstrated the shedding of *C. burnetii* in *I. ricinus* faeces, suggesting its potential role as a reservoir in the wild [27]. In the studied area, *I. ricinus* and wild ungulates do not appear to have a pivotal role in *C. burnetii* maintenance in the wild. To date, their role in the sylvatic cycle has not yet been clarified: however, several cow and goat farms were found to be positive in the same regions [57, 58].

Similarly, all blood and tick samples were negative for TBEV based on direct identification, in line with the role of wild ungulates as tick hosts instead of reservoirs [34]. Moreover, direct investigation in ticks is a sensitive tool for TBEV identification only when testing high amounts of specimens since the prevalence is usually low. Similar results have been reported by other authors in the same area [23], where the prevalence in questing ticks was 0.21% after testing more than 2300 samples [59]. Therefore, other more cost-effective methods should be considered to assess the risk of new foci. The present study shows the importance of serological surveys, which yielded a high flavivirus seroprevalence (i.e., 30.6%). Most of the samples (26.1%) were positive for antibodies against TBEV (Table 3) and were collected from chamois, red deer, roe deer and wild boar, revealing a high seroprevalence of TBEV in the investigated area. As reported by other authors, wild ungulates can be considered suitable sentinels for TBEV circulation [41, 60]. TBEV antibodies were found - at a lower percentage - in many artiodactyls all over Europe: in roe deer from 2.1 to 22.9% [40, 43, 60, 61]; in wild boar from 5.6 to 20% [40, 43, 62]; in moose at 0.74%; in white-tailed deer at 0.74% [42]; and in red deer at 1.4% [41]. Only Krzysiak et al. (2021) found a higher seroprevalence in European bison (62.7%) [34].

Comparing the different seroprevalence values, a great influence of the investigated area seems in place. Knowing that TBEV foci are localized and not homogeneous, a different pattern between and within nations can be expected. In fact, when sampling a larger area, a lower prevalence is expected, and when sampling smaller areas, high variability is likely [41, 60]. The present study focused on two of the most affected areas in Italy for TBEV; thus, a higher seroprevalence was expected compared to other Italian regions. However, the investigated region is considered a low-risk area in the European context, and the remarkable seroprevalence described is in contrast with the scarce human reports, suggesting probable under reporting of cases [20].

Imhoff et al. (2015) highlighted some critical aspects of the use of TBEV serological techniques as screening methods, such as haemolysis of sera (related to the shot) and the scarce precision of the geographical data, due to the wide foraging area of wild ungulates [36]. Despite haemolysis in some samples, antibody detection was confirmed by two different diagnostic tests: ELISA and VNT, the gold standard test. As proposed by other authors, precise testing of TBEV antibodies in wild ungulates could be useful to establish risk maps in areas where data based only on human incidence could be biased, considering the high human TBEV vaccination coverage [60, 61]. Indeed, finding positive animals in areas where no human cases were previously reported would be important for the identification of other potential risk areas [24, 60]. Of note, TBEV seroconversion in alpine chamois has never been described in Europe before. This alpine species could be relevant for the early detection of new TBEV foci as an indirect sentinel of positive ticks in areas where other ungulate species are less frequently observed.

The results of the VNTs also highlighted the presence of USUV and WNV antibodies, detected in four wild boars (USUV – 3.6%) and one roe deer (WNV – 0.9%). The preference of *Culex* mosquitos for wild boars rather than ruminants, when present in the same area, was thus confirmed as already proposed [43]. USUV was known to be present in the studied region: in fact, a human case has been recently described [63], but it had never been identified in wild boars before [32]. Few studies researched USUV antibodies in wild boars and found similar (3.4% in Serbia) or higher (8% in France) seroprevalences [43, 64].

Regarding WNV antibodies in roe deer, both higher (23.5% in Serbia, 4.8% in Czech Republic) and lower seroprevalence (0% in Spain) were described [44, 64, 65]. The positive roe deer identified in the present study were found in Belluno Province, where until 2020, no human cases were reported [33]. West Nile disease cases were mainly located along the Po Valley and the description of WNV at northern latitudes may reflect the effects of climate change.

## Conclusion

When dealing with vector-borne diseases, all epidemiological data may be useful to better understand their prevalence and diffusion. Emerging pathogens are often identified in vectors and/or animals first, and only later the same pathogens are diagnosed in humans [29, 33, 35, 66, 67]. Emerging infectious agents such as *N. mikurensis* and *Ca.* R. mendelii were identified, but they have never been diagnosed in humans in the studied area; thus, their presence should be acknowledged going forward. Tick sampling from wild ungulates has proven effective for tick-borne pathogen surveillance, as well as serological surveys on wild ungulates, due to the remarkable susceptibility of these wild species to flaviviral infections. Further epidemiological studies should take these aspects into account, since wild ungulate monitoring could serve as an early warning system for the detection of viral diffusion in areas considered lacking but at risk of vector, both mosquito and tick, expansion.

## Methods

### Area description

**Fig. 1:**
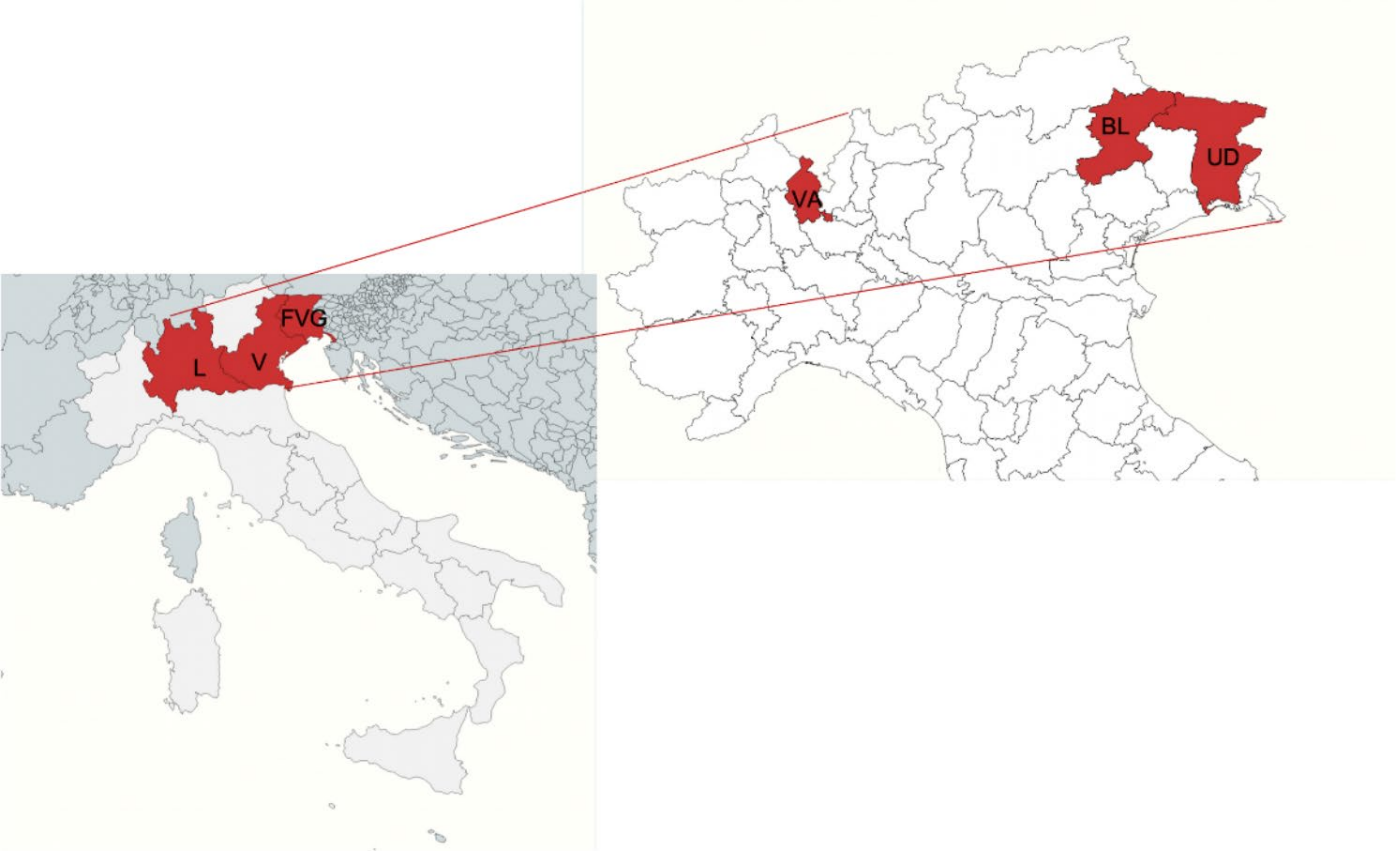
Descriptive map highlighting the study area of sample collection. On the left, the three investigated regions (FVG, V, L) are red. On the right, a detailed image highlights the three provinces where sampling procedures were carried out, Udine (UD), Belluno (BL) and Varese (VA). Map created with mapchart.net [73] and modified with Microsoft PowerPoint (Microsoft office 365)

The study area encompassed the Friuli Venezia Giulia (FVG), Veneto (V), and Lombardy (L) regions, located in northern Italy (Fig. 1). Prealpine and alpine areas located in the provinces of Udine (UD), Belluno (BL) and Varese (VA) were investigated. These areas are approximately located at latitudes between 45°-46° N and are characterized by a wide altimetric excursion, ranging from 180 m a.s.l. in hilly areas up to more than 3000 m a.s.l. of the highest mountain peaks. The climate of the area under study is characterized by the climatic features of the alpine region with relevant temperature excursions between seasons in relation to different altitudes. Higher altitudes are characterized by continental weather: winter months are cold and snowy, while the temperature is mild during warm seasons. In the prealpine area, the climate is overall milder, and during the hunting seasons, the temperatures range from 0 °C to approximately 30 °C, with a maximum humidity reaching 70–98% in several periods (data extrapolated from ARPA Veneto, ARPA FVG and kindly provided by ARPA Lombardia) [68–70].

In addition, the alpine region is facing continuous and dramatic changes due to global warming, which highlights not only a change in temperature but also several other modifications regarding snow cover, humidity, precipitation, vegetation and natural hazards [71, 72].

### Sample collection

Sampling procedures were carried out between May 2017 and September 2020 during licenced hunting seasons. Blood samples were collected by hunters from wild ungulates in 9 mL Vacumed® tubes with K_3_EDTA (FL Medical srl, Italy) in hunting check stations or directly in the field. Ungulate carcasses were carefully inspected for ectoparasites by hunters and/or a veterinarian. When present, ticks were collected up to a maximum of 10 specimens per carcass, and each of them was placed in a 1.5 mL sterile microtube. An anamnestic form was filled out for each animal recording species, age, sex, date and place of culling, weight, health condition, and presence/absence of ticks. Both blood and tick samples were stored at 4 °C and sent to the Laboratory of Microbiology and Infectious Diseases of the Animal Medicine, Production and Health Department (Legnaro, Italy). Once in the laboratory, blood samples were divided into 200 µL aliquots while the ectoparasites were first morphologically identified using the identification keys of Manilla and Cringoli [74, 75] and then placed, individually or in pools of a maximum of two specimens, in a new 1.5 mL sterile microtube. Pools included ticks from the same host with the same features (species, sex, stage), and a low engorgement stage. Both blood samples and ectoparasites were stored at -80 °C until processing.

### Molecular analyses

Nucleic acids were extracted from 200 µL of whole blood and tick samples using the All Prep DNA/RNA Mini Kit (QIAGEN GmbH, Germany) following the manufacturer’s instructions. Each tick sample was ground thoroughly with a sterile disposable plastic pestle in a 1.5 mL microtube, resuspended in 350 µL of Buffer RLT Plus, and homogenized by repetitive pipetting.

Before the lysis step during nucleic acid extraction, both DNA and RNA internal controls, supplied by the Quantinova Pathogen + IC kit (QIAGEN GmbH, Germany), were added to all specimens to assess both extraction efficiency and the presence of PCR inhibitors. Extracted nucleic acids were stored at -80 °C (RNA) or -20 °C (DNA) and then screened using previously published PCR and real-time PCR methods to detect the following viral and bacterial pathogens: TBEV [76], *B. burgdorferi* s.l. [77, 78], *Rickettsia* spp. [79, 80], *Coxiella* spp. [81], *Ehrlichia* spp. [82] and *N. mikurensis* [83, 84]. Positive and negative controls were included in each run. Details about the methods and procedures are provided in Additional file 1.

Internal control detection and pathogen screening were performed using a Quantinova Pathogen + IC kit (QIAGEN GmbH, Germany) on a LightCycler96 Instrument (Roche, Switzerland) with the Internal Control Assay kit and genus-specific real-time PCR assays (Additional file 1). *N. mikurensis* HRM real-time PCR assays were performed using 5x HOT FIREPol EvaGreen qPCR Mix Plus (Solis Biodyne) on a MyGo Pro instrument (IT-IS, United Kingdom). *Ehrlichia* spp. endpoint PCR screening was performed using 1× Phire Hot Start II PCR Master Mix (Thermo Fischer Scientific Baltics, Lithuania) on a Biometra TGradient thermal cycler (Analytic Jena GmbH, Germany).

All samples yielding a positive signal in both the internal control assay and pathogen screening were further investigated by specific end-point PCR assays (Additional file 1), followed by Sanger sequencing of amplicons. PCRs were performed with the same reagents and instrument used for *Ehrlichia* spp. screening.

PCR products were visualized by electrophoresis on 2% agarose gels stained with SybrSafe DNA Stain (Invitrogen by Thermo Fischer Scientific, USA) and subsequently purified using ExoSap-IT Express PCR Product Cleanup (Thermo Fischer Scientific Baltics, Lithuania) according to the manufacturer’s instructions. Bidirectional Sanger sequencing of all specific PCR products was carried out at the StarSEQ® GmbH facilities (Mainz, Germany) using the same PCR primers. Nucleotide sequences were assembled and edited using ChromasPro v.2.1.8 (Technelysium Pty Ltd, Australia) and were then deposited in GenBank (Accession numbers in Additional file 2) and analysed using the Nucleotide BLAST [85] search engine (National Center for Biotechnology Information, Bethesda, MD).

### ELISA and virus neutralization for flaviviruses

Serum samples were collected in 9 mL Vacumed® tubes without anticoagulants (FL Medical srl, Italy). To assess the seroprevalence of TBEV, serum samples were screened with the two-step ELISA test Immunozym FSME IgG all species (PROGEN, Biotechnik GmbH, Heidelberg, Germany). According to the manufacturer’s recommendations, sera were diluted 1:50. The results of the test were expressed in Vienna International Units and samples were considered positive with > 126 Vienna units/mL, borderline if between 63 and 126 Vienna units/mL, and negative when < 63 Vienna units/mL. Positive and borderline samples were further tested with the gold standard confirmatory test, i.e., the virus neutralization test. VNTs were performed at the National Reference Laboratory for Arboviruses of the Ostrava Public Health Institute (Ostrava, Czech Republic). Anti-TBEV VNTs were performed using sterile 96-well plates. The TBEV strain (Hypr) was cultivated in intracerebrally infected suckling mice, and PS cells (porcine stable kidney cell line) were used as the susceptible cell line; VNTs were performed and the results were expressed as previously described [86] with minor changes consisting of the use of 25 µL of PS cell suspension (600,000 cells per mL) instead of the CV-1 cell line (African green monkey kidney fibroblasts). For anti-WNV and anti-USUV tests, an identical procedure was used, choosing the CV-1 cell line as the susceptible cell line for both viruses. WNV lineage 2 and USUV lineage Eur3 were used as virus suspensions. In each VNT, the endpoint titre was assessed as the higher serum dilution that inhibited the viral cytopathic effect. Samples showing a titre of anti-Flavivirus antibodies equal to 1:8 or higher were considered positive.

### Statistical analysis

Data regarding sample collection and laboratory analyses were organized in a database on a Microsoft Excel Worksheet and descriptive statistics (counts, percentage and CI95%) were used to summarize results according to pathogen detection or serological results with respect to “species”, “sex”, and “age” of the animals, and “season” and “region” of the collection variables; the statistical analyses to detect significant differences in infection rates were conducted by means of chi-square test or Fisher’s exact test, when appropriate. The level of statistical significance was set for alpha = 0.05.

### Electronic supplementary material

Below is the link to the electronic supplementary material.

Supplementary Material 1

Supplementary Material 2

Supplementary Material 3

## Data Availability

All data generated or analysed during this study are included in this research article [and its additional files 1, 2, and 3]. Further information may be available from the corresponding author upon request.

## Abbreviations

*A. phagocytophilum*: *Anaplasma phagocytophilum*
*B. burgdorferi* s.l.: *Borrelia burgdorferi* sensu lato
*B. garinii*: *Borrelia garinii*
*B. afzelii*: *Borrelia afzelii*
*B. burgdorferi* s.s.: *Borrelia burgdorferi* sensu stricto
*B. valaisiana*: *Borrelia valaisiana*
BL: Belluno
*Ca.* R. mendelii: *Candidatus* Rickettsia mendelii
*C. burnetii*: *Coxiella burnetii*
FVG: Friuli Venezia Giulia
*I. ricinus*: *Ixodes ricinus*
L: Lombardy
m a.s.l.: metres above sea level
*N. mikurensis*: *Neoehrlichia mikurensis*
*R. monacensis*: *Rickettsia monacensis*
*R. helvetica*: *Rickettsia helvetica*
TBDs: Tick-borne diseases
TBPs: tick-borne pathogens
TBEV: tick-borne encephalitis virus
UD: Udine
USUV: Usutu virus
VA: Varese
V: Veneto
VNT: virus neutralization test
WNV: West Nile virus
