# Supplementary material for: Wild ungulates as sentinels of flaviviruses and tick-borne zoonotic pathogen circulation: an Italian perspective
Source: BMC Vet Res. 2023 Sep 14;19:155. doi: 10.1186/s12917-023-03717-x (PMC10500747; doi:10.1186/s12917-023-03717-x)
Supplement: Supplementary file 3 — Supplementary Material 3 [file 12917_2023_3717_MOESM3_ESM.pdf]

Additional file 1. Biomolecular methods used for TBPs screening and sequencing, detailed information about bacterial/viral species, PCR method used, target gene, primers, nucleotide sequence and probes, amplicon size and bibliography references.

Species	PCR method	Target gene	Primer	Nucleotide sequence 5' → 3'	Amplicon	Reference
Ehrlichia spp.	PCR	groEL	groEL643f	ACTGATGGTATGCARTTTGAYCG	600-bp	(1)
			groEL1236r	TCTTTRCGTTCYTTMACYTCAACTTC		
Neoehlichia mikurensis	HRM Real-time PCR	16S rRNA	F	GGGGATGATGTCAARTCAGCAY	257-bp	(2)
			R	CACCAGCTTCGAGTTAAGCCAAT		
	PCR	16S rRNA	fD1	AGAGTTTGATCCTGGCTCAG	760-bp	(3)
			Ehr16SR	TAGCACTCATCGTTTACAGC		
Borrelia b. s.l.	Real-time PCR	23S rRNA	Bb23Sf	CGAGTCTTAAAAGGGCGATTTAGT	75-bp	(4)
			Bb23Sr	GCTTCAGCCTGGCCATAAATAG		
			Bb23Sp-FAM	AGATGTGGTAGACCCGAAGCCGAGTG		
	PCR	groEL	GF	TACGATTTCTTATGTTGAGGG	310-bp	(5)
			GR	CATTGCTTTTCGTCTATCACC		
Rickettsia spp.	Real-time PCR	gltA	RKND03f	GTGAATGAAAGATTACACTATTTAT	-	(6)
			RKND03r	GTATCTTAGCAATCATTCTAATAGC		
			RKND03-FAM	CTATTATGCTTGCGGCTGTCGGTTC		
	Nested PCR	gltA-outer	RpCS.877p	GGGGGCCTGCTCACGGCGG	381-bp	(7)
			RpCS.1258n	ATTGCAAAAAGTACAGTGAACA		
	Nested PCR	gltA-inner	RpCS.896p	GGCTAATGAAGCAGTGATAA	337-bp	(7)
			RpCS.1233n	GCGACGGTATACCCATAGC		
Coxiella spp.	Real-time PCR	icd	icd-439f	CGTTATTTTACGGGTGTGCCA	76-bp	(8)
			icd-514r	CAGAATTTTCGCGGAAAATCA		
			icd-464-FAM	CATATTCACCTTTTCAGGCGTTTTGACCGT		
TBEV	Real-time PCR		TBE-f	TGGGCGGTTCTTGTTCTCC	-	(9)
			TBE-r	TCACACATCACCTCCTTGTCAGA		
			TBE-FAM	CTGAGCCACCATCACCCAGACACAG		

References

1. Guillemi EC, Tomassone L, Farber MD. Tick-borne Rickettsiales: Molecular tools for the study of an emergent group of pathogens. J Microbiol Methods. 2015;119:87–97.

2. Krücken J, Schreiber C, Maaz D, Kohn M, Demeler J, Beck S, et al. A novel high-resolution melt PCR assay discriminates Anaplasma phagocytophilum and "Candidatus Neoehrlichia mikurensis." J Clin Microbiol. 2013;51(6):1958–61.

3. Goodman JL, Nelson C, Vitale B, Madigan JE, Dumler JS, Kurtti TJ, et al. Direct Cultivation of the Causative Agent of Human Granulocytic Ehrlichiosis. N Engl J Med. 1996;334(4):209–15.

4. Courtney JW, Kostelnik LM, Zeidner NS, Massung RF. Multiplex real-time PCR for detection of Anaplasma phagocytophilum and Borrelia burgdorferi. J Clin Microbiol. 2004;42(7):3164–8.

5. Lee SH, Lee JH, Park HS, Jang WJ, Koh SE, Yang YM, et al. Differentiation of Borrelia burgdorferi sensu lato through groEL gene analysis. FEMS Microbiol Lett. 2003;222(1):51–7.

6. Rolain JM, Bitam I, Buffet S, Marié JL, Bourry O, Portelli-Clerc C, et al. Presence or absence of plasmid in Rickettsia felis depending on the source of fleas. Eur Soc Clin Microbiol Infect Dis. 2009;15 Suppl 2:296–7.

7. Choi YJ, Jang WJ, Kim JH, Ryu JS, Lee SH, Park KH, et al. Spotted fever group and typhus group rickettsioses in humans, South Korea. Emerg Infect Dis. 2005;11(2):237–44.

8. Klee SR, Tyczka J, Ellerbrok H, Franz T, Linke S, Baljer G, et al. Highly sensitive real-time PCR for specific detection and quantification of Coxiella burnetii. BMC Microbiol. 2006;6:1–8.

9. Schwaiger M, Cassinotti P. Development of a quantitative real-time RT-PCR assay with internal control for the laboratory detection of tick borne encephalitis virus (TBEV) RNA. J Clin Virol. 2003;27(2):136–45.
